# Computational modeling of rhythmic expectations: Perspectives, pitfalls, and prospects

**DOI:** 10.1371/journal.pcbi.1013798

**Published:** 2025-12-10

**Authors:** Atser Damsma, Jonathan Cannon, Lauren K. Fink, Keith B. Doelling, Jessica A. Grahn, Henkjan Honing, Thomas Kaplan, Edward W. Large, Fleur L. Bouwer

**Affiliations:** 1 Music Cognition Group, Institute for Logic, Language, and Computation, Amsterdam Brain and Cognition, University of Amsterdam, Amsterdam, The Netherlands; 2 Conservatorium van Amsterdam, Amsterdam University of the Arts, Amsterdam, The Netherlands; 3 Department of Psychology, Neuroscience & Behaviour, McMaster University, Hamilton, Ontario, Canada; 4 Université Paris Cité, Institut Pasteur, AP-HP, INSERM, CNRS, Fondation Pour l’Audition, Institut de l’Audition, IHU reConnect, Paris, France; 5 Department of Psychology & Centre for Brain and Mind, Western University, London, Ontario, Canada; 6 William Harvey Research Institute, Barts and the London Faculty of Medicine and Dentistry, Queen Mary University of London, London, United Kingdom; 7 Barts Biomedical Research Centre, National Institute of Health and Care Research, Queen Mary University of London, London, United Kingdom; 8 Center for the Ecological Study of Perception and Action, Department of Psychological Sciences and Department of Physics, University of Connecticut, Storrs, Connecticut, United States of America; 9 Cognitive Psychology Unit, Institute of Psychology & Leiden Institute for Brain and Cognition, Leiden University, Leiden, The Netherlands; Basque Center on Cognition Brain and Language, SPAIN

## Abstract

Rhythmic structure enables precise temporal expectations that are essential to human communication, including speech and music. Computational models have been developed to account for how humans perceive, produce, and learn rhythmic sequences. However, it is unclear how different types of models relate to each other and how they can be evaluated. In this review and perspective, we discuss how three major classes of models—entrainment, probabilistic, and timekeeper models—have been used to study rhythmic expectations. We critically assess each model class in terms of its level of explanation, the rhythmic behaviors it captures, its ability to account for learning and enculturation, and its ability to integrate other features, such as pitch. We show that entrainment, probabilistic, and timekeeper models differ substantially in the aspects of rhythmic expectations they can capture. To move the field forward, we propose that model comparison and integration are crucial. We identify key challenges to this effort, such as the varying nature of the input and output signals and divergent modeling goals. To address these challenges, we arrive at several practical recommendations: to equate input and output signals when comparing models, to consider several model outcomes beyond goodness-of-fit measures in model evaluation, to use model-integration efforts to inform theory building, and to make code and data openly accessible. Ultimately, understanding how models of rhythmic expectations relate, and how features in these models account for behavioral, neural, and cognitive aspects of rhythmic expectations, will deepen our understanding of a core aspect of human behavior.

## 1. Introduction

In our dynamic environment, events unfold over time. The way events are structured in time is commonly referred to as *rhythm* [1]. Rhythmic structure allows us to predict event timing, and thereby, to optimize processing [2,3]. Predictable rhythms allowing for precise temporal expectations are found in many natural phenomena and signals, but are especially prominent in language and music. In music, rhythmic expectations have been linked to reward and pleasure [4], social bonding [5], and movement facilitation [6].

Computational modeling is an essential tool in understanding rhythmic behavior and cognition, aiming to describe the mechanisms underlying rhythmic expectations. Computational models formalize theories of human cognition beyond the verbal level to concrete mathematical or programmatical formulations that can be tested against behavioral and neural data [7]. This has important advantages: first, it enables us to directly compare models representing competing theories [7–9]. For example, comparing competing computational models to human data can elucidate whether synchronization relies on estimating concrete durations (*absolute timing*) or on timing relative to a regularity or a previous duration (*relative timing*) [10,11]. It could also shine light on competing theories about the neural networks supporting rhythm perception, such as the Vocal Learning [12] and Gradual Audiomotor Evolution [13] hypotheses. Second, modeling allows for parameter manipulation to explain individual differences meaningfully [14]. Third, in-silico ‘lesioning’ allows for a systematic examination of how performance is affected by model components, mimicking how brain pathology affects behavior [7].

Different types of computational models capture how listeners perceive and produce rhythm, but it is unclear how these models may be related [15,16] and evaluated [8], hampering our understanding of rhythmic expectations. In this critical review and perspective, we will therefore consider the current state of the field and explicitly address ways to move it forward.

In our discussion, we use the term *rhythm* to refer to any sequence of multiple temporal intervals, including various types of non-isochronous patterns. We focus on models capturing rhythm processing in real time, as it unfolds, excluding models that can only consider a full rhythmic pattern at once [17]. Moreover, we only include models of cognition and brain activity, which formalize some theoretical account [7] of how listeners form rhythmic expectations. This excludes generative AI models [18], which, while arguably successful at processing musical signals, have not yet explicitly contributed to understanding human rhythm cognition. Finally, we focus on models of non-linguistic rhythm. While rhythm is essential to language, linguistic rhythm differs from musical rhythm in several ways. First, whereas periodicity plays an important role in non-linguistic rhythm, it has been argued that language is only quasi-periodic [19]. Second, the semantic content of language may influence its rhythmicity [19,20]. Third, while we focus on rhythm as continuous timing, research on linguistic rhythm typically emphasizes lexical stress patterns and grouping [19].

**Fig 1:**
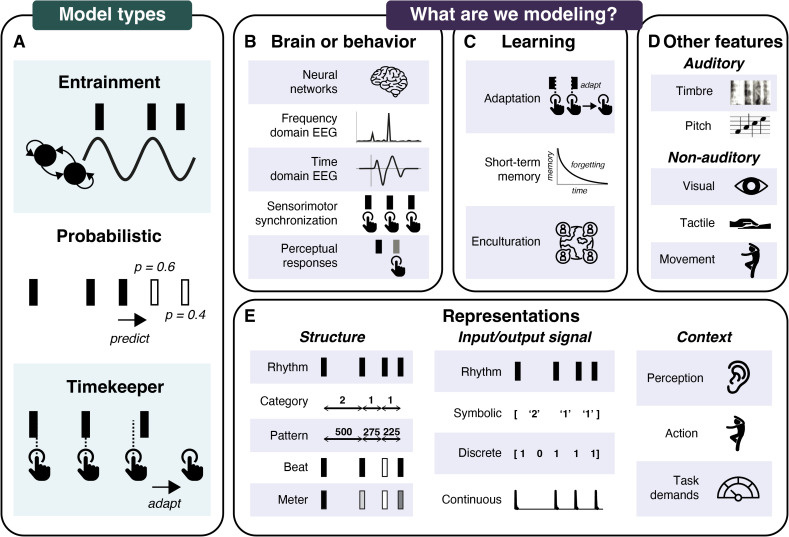
Schematic overview of three classes of models (Section 2), and their targets in terms of modeling (Section 3). Different models (**A**, Section 2) operate at different levels of explanation (**B**, Section 3.1.), and target different rhythmic representations (**E**, Section 3.2.). Models also diverge in their treatment of learning (**C**, Section 3.3.) and non-temporal or non-auditory features (**D**, Section 3.4.). Icons were licensed through *Icon Pro* from *The Noun Project* (https://thenounproject.com/). Spectrogram in Fig 1D by Aaron Parecki (https://www.flickr.com/photos/aaronpk/4947807970) [CC BY 2.0].

Given these constraints, we discuss three broad classes of models (Fig 1A): entrainment, probabilistic, and timekeeper models. These three classes capture the current state-of-the-art in modeling rhythm processing dynamically, as time unfolds [1]. We first introduce each model class and their specific implementations (see Table 1 for a non-exhaustive overview). We then show how these classes differ fundamentally in their level of explanation, the rhythmic representations and learning processes they capture, and their integration of features beyond auditory timing (Fig 1B–1E). Entrainment models take neural mechanisms as their starting point and have been linked to neural markers of periodicity, such as beat and meter; probabilistic models are rooted in cognitive theory and typically capture behavioral markers of symbolic sequences prediction; and timekeeper models emphasize isochronous motor synchronization. Given these discrepancies, we discuss strategies to move the field forward through model comparison and integration and offer practical recommendations for future modeling studies on rhythmic expectations.

**Table 1:** Overview of different models of rhythmic expectations.

Model class	Type of model	Data domain	Phenomenon studied
Entrainment	Bank of linear oscillators	Continuous time	Beat, meter [21–24]; Groove [23]; Sensorimotor synchronization [23,24]
	Multiple connected banks of coupled nonlinear oscillators	Continuous time	Beat, meter [25–28]; (Onset) Pattern [26]; Enculturation [26]; Groove [28]
	Multiple coupled nonlinear oscillators	Continuous time	(Quasi-)Isochrony [29–31]; Beat, meter [32–34]; Interpersonal synchronization [29–31]; Auditory-motor interactions [34]; Expressive timing [33]; Spontaneous motor tempo [31]
	Single nonlinear oscillator	Continuous time	(Quasi-)Isochrony [35–39]; Beat, meter [35,40]; Interpersonal synchronization [38]; Gait [37]
Probabilistic	PPM (prediction by partial matching); Markov chains; Bayesian	Symbolic/categorical	Meter [41,42]; (Onset) Pattern [11,43–46]; Enculturation [41]; Groove [47]
	Phase inference from point process event timing (PIPPET)	Continuous time	(Quasi-)Isochrony [48–50]; Beat, meter [48–50]; (Onset) Pattern [51]; Meter [51]; Enculturation [51]; Expressive timing [50]
	Phase inference combined with PPM	Continuous time	Beat, meter, pattern [52]
Timekeeper	Timekeeper with error correction	Continuous value	(Quasi-)Isochrony [53–55]; Interpersonal synchronization [53,55]
	Timekeeper with error correction and anticipation	Continuous value	(Quasi-)Isochrony [56–59]; Interpersonal synchronization [56–58]
Hybrid	Single nonlinear oscillator with probabilistic input for period adjustment	Continuous time	(Quasi-)Isochrony [10]
	Single nonlinear oscillator with added phase/period correction	Continuous time	(Quasi-)Isochrony [60]

**Note:** This table provides a non-exhaustive overview of models, to illustrate their diversity, and the various domains and rhythmic phenomena studied. Data domains are divided into continuous time (a value outputted at every time point, e.g., a continuous time signal), continuous value (a value outputted on a continuous scale, e.g., the next expected IOI on a continuous scale) or symbolic (a symbolic value as output, e.g., the next expected IOI from a limited set of possible IOIs). Concerning the phenomena studied: we use the term “(quasi-)isochrony” to denote not just isochronous sequences (e.g., metronomes), but also sequences derived from isochrony, with tempo or phase perturbations, or variability introduced. We consider these quasi-isochronous, as they do not explicitly contain higher order structure as found in beat and meter.

## 2. Models capturing rhythmic expectations

### 2.1. Entrainment models

Human music often contains a high degree of (quasi-)periodicity, which is perceived by listeners as a regular *beat*. Entrainment models capitalize on this property of musical rhythm. Broadly, *entrainment* refers to the phase alignment between two periodic signals. In theories of rhythmic expectations, entrainment involves a (quasi)periodic process in the nervous system—such as low-frequency cortical oscillations—becoming phase-aligned with external rhythmic input [61]. Cognitively, this alignment may cause fluctuations in perceptual performance [2]. Motorically, entrainment may lead to the synchronization of movement with rhythmic input or coordination between multiple individuals [15,29].

Computational models of entrainment typically assume an (endogenous) oscillator which adapts its phase and period to an external regularity, but vary significantly in their properties. First, some models, such as the Beyond-the-Beat model, use linear oscillators, which essentially act as filters [21,24]. Others employ non-linear oscillators, which enable *mode-locking*, with entrainment occurring at frequencies that are related to the eigenfrequency of the oscillator at integer ratios [1]. Second, the flexibility of an oscillator to adapt to frequencies other than its eigenfrequency can be manipulated [40]. Third, whether oscillators are self-sustaining depends on the choice of damping parameters [39,62]. Fourth, the strength of phase coupling between the external stimulus and the oscillator further determines how quickly and robustly entrainment occurs [30,62]. Finally, instead of relying on a single oscillator, many entrainment models—including the Beyond-the-Beat model and gradient frequency neural networks—employ a bank of oscillators reflecting the frequency range of human rhythm perception [21,22,24,27]. The behavior of these models depends on the number of oscillators, their center frequencies, and whether oscillators are interconnected, with phases either coupled [25] or uncoupled [26]. More complex architectures may include interconnected networks of oscillators, mimicking the interaction between auditory and motor systems [1,63].

### 2.2. Probabilistic models

Probabilistic models are often situated within the predictive coding framework [64,65]. This framework proposes that the brain constantly generates predictions of sensory input based on a probabilistic mental model of the world. These top-down predictions are compared against the actual input, and prediction errors are used to update the mental model in an approximation of Bayesian inference. In the domain of rhythm perception, probabilistic models aim to uncover the temporal structure that underlies a sequence of sounds (i.e., the mental model), to predict the timing of future events (Fig 1A). Probabilistic models can provide event-by-event measures such as *surprisal* (how unexpected an event is) and *uncertainty* (the precision of the prediction), which have been shown to match human ratings of rhythmic complexity [44,47]. Through Bayesian updating, rhythmic expectations can be revised dynamically in response to new sensory information. Additionally, long-term listening experiences can be encoded as *priors* [41,42,47,51].

Like entrainment models, probabilistic models vary in their computational properties. Models based on IDyOM (Information Dynamics of Music) [42] represent rhythm as a sequence of symbols, with transitional probabilities between them learned using Markov chains [11]. In these models, the *order-bound* parameter determines the maximum length of history (context) the model is allowed to consider when predicting the next symbol. Recently, an alternative was proposed with PIPPET (Phase Inference from Point Process Event Timing), which formalizes rhythm perception as a phase inference problem [48] and functions in continuous time. This model tracks phase within a sequential or cyclical ‘expectation template’: a predefined [48] or inferred [51] representation of an anticipated (metrical) pattern. The event likelihood precisions determine the sharpness of these temporal expectations. The estimated phase and its uncertainty are adjusted based on incoming events, where the accumulation of temporal uncertainty is governed by the diffusion rate. Interestingly, a cyclical version of the model [49] shows properties that are mathematically equivalent to a damped oscillator, suggesting that probabilistic and entrainment models may align functionally.

### 2.3. Timekeeper models

Timekeeper models use a linear autoregressive process to model the timing of an event based on the time interval and asynchrony associated with the previous event (Fig 1A) [58]. These models propose an error-correction mechanism to account for timing and motor variability, relying on two separate mechanisms: period and phase correction [53]. The magnitude of these corrections is controlled by the period correction rate and phase adaptation rate, respectively [58]. One implementation, the Adaptation and Anticipation Model (ADAM), also includes an anticipation process [56,58] in which upcoming events are anticipated through linear extrapolation of previous intervals [59]. Anticipation is parameterized by the number of intervals used for extrapolation and the prediction-tracking balance. In addition, the weighting between reactive adaptation and anticipation can be adjusted [56].

More recently, a neuro-mechanistic model was proposed in which a biophysically-based beat generator neuron (BG) synchronizes with a (quasi)isochronous sequence [60]. The period and phase of the BG are adjusted based on a comparison between the number of gamma-frequency cycles between two successive BG spikes and a similar cycle count between stimulus onsets. As such, the BG model can be seen as a neuronal implementation of the error-correction mechanisms that characterize timekeeper models.

## 3. What are we trying to model? A critical comparison of existing models

### 3.1. Behavior or brain mechanisms

While the models discussed above are all implemented algorithmically [66], they operate at different explanatory levels (Fig 1B). Probabilistic models mainly account for behavioral or neural markers that indirectly index rhythmic expectations, such as event-related potentials (ERPs) [67,68], without explicitly specifying the underlying neural processes. General predictive processing models hypothesize that higher-level brain areas provide predictions to lower-level areas via feedback connections, with lower-level areas encoding prediction errors [65]. However, these models often focus on *content* expectations (i.e., predicting “what”, see [69]) instead of *temporal* expectations (i.e., predicting “when”), raising questions about the relevance of this neural model for rhythm prediction. Notably, a recent model based on the Action Simulation for Auditory Prediction (ASAP) hypothesis does attempt to specify the neural networks underlying rhythm processing—including the motor system [70], but this model currently lacks an algorithmic implementation.

In contrast to probabilistic models, entrainment models, like those based on neural resonance theory [1], take the neural level as their starting point. Aspects of behavior emerge from well-studied neural mechanisms (e.g., neuronal oscillation, Hebbian plasticity, synaptic transmission delay, see [1]), instead of being directly implemented. For example, *negative mean asynchrony*—the tendency to tap earlier than note onsets when synchronizing to rhythm [71]—may be parsimoniously explained as a generic effect of neural transmission delays [31,38,72]. Entrainment models of rhythmic expectations have been tested against both neural and behavioral data. They can mimic the selective enhancement of power at beat frequencies in response to non-isochronous rhythm found in human EEG data (*frequency tagging*, see [25]) and motor synchronization [25]. However, frequency tagging as evidence for neural entrainment should be interpreted cautiously, as it may alternatively reflect ERPs to rhythmic stimuli [73,74] or sequential anticipatory neural activity [75].

Timekeeper models have mostly been applied to behavioral sensory-motor synchronization (SMS): the coordination of movement with an external rhythm [71], be it the synchronizing of finger tapping to rhythm, adaptive synchronization behavior between two humans [76], or between humans and “virtual partners” [77]. One fMRI study has associated ADAM’s adaptation and anticipation parameters to specific brain areas, including the cerebellum, basal ganglia, and thalamus [57], linking the behavioral to the neural level.

### 3.2. Types of representation

#### 3.2.1. Types of structure.

In addition to differences in explanatory level, models handle the inherent multiplicity of rhythmic structure (Fig 1E) in different ways. Listeners infer structure from rhythmic sequences by learning predictable *patterns* (i.e., recurring successions of longer and shorter intervals [11]), and by inferring a *beat* (i.e., isochronous pulses) and *meter* (i.e., a hierarchical organization of beats [1]). While these processes may interact [51,78], evidence from behavioral [79] and neural studies [80] suggests they are at least partially dissociable.

Many models exclusively target (quasi-)isochronous sequences. Isochrony processing is often studied by examining reactions to timing perturbations [10,29,30,36,37,39,54,58]. Human responses to perturbations have successfully been modeled using timekeeper models with explicit parameters for phase and period correction [58], and networks of oscillators, like in research examining interpersonal synchrony [30], where the complexity of multiple oscillators was necessary to explain human behavior. Additionally, human temporal fit judgments after quasi-isochronous sequences were modeled using a hybrid adaptive oscillator model, in which a probabilistic aspect was added to a single eigenfrequency entrainment model to account for timing variability in the stimulus [10]. Various classes of models have thus been successful in explaining human responses to (quasi-)isochronous sequences. However, since pattern, beat, and meter may be simultaneously present in isochronous sequences, it is unclear what rhythmic percept these models target. Also, they may not handle complex non-isochronous sequences well, as is apparent for some timekeeper models [40].

Several probabilistic models have explicitly targeted rhythmic patterns [11,43,44,46,51]. Markov-chain models using symbolic representations of absolute inter-onset intervals (IOIs) were used to predict complexity ratings [44] and tapping accuracy [43] in response to rhythmic patterns. Rather than absolute IOIs, other probabilistic models have used representations of ratios between successive intervals [46,51]. While probabilistic models may naturally fit pattern learning, non-isochronous rhythmic patterns can also be learned by an entrainment model using Hebbian plasticity [26]. Here, patterns could be categorized based on oscillator amplitude at different frequencies. However, the input sequences used also contained hierarchical structure represented by loudness differences between sounds, making it unclear whether the output only reflected the temporal features of the pattern.

Hierarchical structure (i.e., meter) has been explicitly targeted by probabilistic models estimating the probability of a sounded event at different metrical positions [41,47]. Here, the meter needs to be known beforehand [47], or the model infers the most likely meter from a set of candidates based on the observed rhythmic pattern [41]. This is similar to PIPPET, which uses cyclical expectation templates [48]—matching the cyclic nature of meter [49]—to predict rhythmic patterns. This type of model has the potential to integrate probabilistic predictions of both rhythmic patterns and meter. However, arriving at such integrated expectations likely requires a bootstrapped learning process, since meter must be inferred from the rhythmic pattern, while the inferred meter is, in turn, used to predict pattern onsets.

Entrainment models often specifically target beat and meter induction. The perceived metrical structure is deduced from the spectral content of the model’s output across an entire rhythmic sequence, with the most prominent frequency assumed to represent the perceived beat [24,25,27]. This raises the question how these models deal with non-periodic meters, which are common in many non-Western musical cultures. Whereas the flexibility of the expectation template in PIPPET explicitly allows for such meters [48], they may be a challenge for entrainment models [81].

#### 3.2.2. Types of input and output signals.

Models differ in the input they accept and the type of output they generate (Fig 1E). Inputs and outputs can be *symbolic* (e.g., categorical representations of temporal intervals [44] or metrical position [41]), or *discrete* (e.g., a binary decision whether a position on a rhythmic grid is filled with an event or not [45,47]), but such representations cannot capture processing of natural rhythms with continuous timing and tempo variations, which would require infinite symbolic event or grid representations. Alternatively, *continuous values* (e.g., the next IOI to be produced with timekeeper models [54,58]), or *continuous time series* have been used to represent fluctuations in rhythmic expectations [10,21,22,25,27]. However, continuous output signals are often reduced to frequency-domain representations for model evaluation, with the largest spectral peak interpreted as the perceived beat frequency [25,27]. This reduction makes it unclear how the model output translates to real-time behavior (e.g., are a listener’s rhythmic expectations indexed by the signal’s amplitude, phase, or both?). Models like PIPPET [48] and the Beyond-the-Beat linear oscillator model [21] avoid this reduction by processing continuous input and producing continuous output.

#### 3.2.3. Perception, action, and task demands.

Both motor synchrony and perceptual advantages at expected time points have been interpreted as reflecting rhythmic expectations. While synchronization and perceptual rhythmic abilities are indeed correlated, and motor behavior strengthens the perceptual effects of rhythmic expectations, perception and action have also been partially dissociated [82,83]. Indeed, how rhythmic expectations are formed may depend on goals and task demands [84], but these are often not explicitly modeled. Timekeeper models have, to our knowledge, been exclusively designed for and applied to motor behavior. This raises the question whether the error-correction mechanisms in timekeeper models also apply to perceptual processing. Probabilistic models have been applied to both production and perception [41,46]. One variable-order Markov model was shown to explain behavior across three different perceptual and tapping tasks within the same participants [11], suggesting that such models index general underlying rhythmic expectations, which influence a variety of behavioral and perceptual markers. Entrainment models have similarly been applied to both perception [21,22,25,26] and action [23,29,31]. Some of these models explicitly contain separate perceptual and motor networks [25]. To study the relationship between perception and action, and the influence of task demands on rhythmic expectations, one approach would be to enable both an active and a passive mode in such models [83]. One study explicitly modeled task demands using a network of coupled oscillators [34], adjusting the coupling strength between perceptual, attention, and motor oscillators to mimic behavioral data under different conditions. Similarly, audio-motor interactions were explicitly modeled to account for the pleasurable urge to move to musical rhythm [28], integrating aspects of rhythmic perception and action.

In summary, the rhythmic aspect, the type of signal, and the type of task that are modeled vary greatly between models. Table 1 provides a (non-exhaustive) overview of model implementations and their targeted rhythmic aspects and signals.

### 3.3. Flexibility and learning

Rhythmic expectations are flexibly shaped by both short- and long-term learning processes (Fig 1C). When synchronizing with a rhythm, humans can rapidly adapt their movement to phase perturbations and tempo-changes [35,39], reflecting crucial short-term flexibility. Entrainment models can likewise adapt to changing regularities within a few cycles [31,40], with the magnitude of adaptation determined by the coupling strength between the rhythmic stimulus and the oscillator [32]. In timekeeper models such as ADAM, adaptation is explicitly incorporated, since the next tapping interval is updated based on the current asynchrony, with separate parameters for phase and period correction [58]. A probabilistic implementation of phase and tempo adaptation can be found in PIPPET, where adaptation is based on recent timing information and depends on uncertainty of the predictions [48,49].

While all of these models thus adapt to small timing perturbations, they do not consider them meaningful rhythmic structure. However, deviations in the order of tens of milliseconds (*microtiming* or *expressive timing*) are common in music performance and may convey relevant rhythmic information on their own, clarifying compositional structure and determining whether a performance feels “pushed” or “laid back” [50]. One oscillator model was shown to sustain synchronization when faced with temporal fluctuations, which interestingly could even improve its tracking ability [33]. Recently, PIPPET was shown to discriminate between drum patterns with variable microtiming profiles, and human individual differences in discrimination were explained by the amount of phase-tracking noise in the model [50]. These efforts stress the importance of modeling rhythm in continuous time to account for microtiming.

Besides flexibility, models need to account for short-term memory. The recent probabilistic PPM-Decay model of auditory sequence prediction implemented an exponential memory decay function, in which the weight of events decreases over time [85], to account for recency biases and capacity limitations in human short-term memory. Memory decay indeed improved prediction of chord sequences [85] and tapping behavior [11]. Timekeeper model ADAM similarly includes an anticipatory module which extrapolates the previous (at least three) taps, essentially acting as a short-term memory buffer for prediction [58]. Likewise, in PIPPET [48], phase uncertainty increases over time, reducing the weight of events as they recede into the past. In entrainment models, oscillator decay time can be seen as an implicit short-term memory parameter [8]. Thus, different models can capture how sequence statistics change over time, as in naturalistic rhythm.

Finally, in production or discrimination tasks, cultural familiarity with the presented rhythms can improve accuracy [86], showing long-term learning. In coupled non-linear oscillator models, Hebbian plasticity has been used to simulate perceptual narrowing [26,63]. Through repeated exposure to specific rhythmic structures, adjustment of coupling coefficients between oscillators improves the network’s response to similar structures. In probabilistic models, such as IDyOM, long-term statistical learning has been implemented by estimating the prior probability of certain rhythmic sequences based on a musical corpus approximating the listening experience of individuals [42]. While such probabilistic models of enculturation typically depend on symbolic representations of musical rhythms, continuous probabilistic phase inference has also been used to simulate enculturation based on the statistical distribution of rhythmic sequences in an empirical sample [51]. Parameter optimization within probabilistic generative models has been directly related to Hebbian plasticity [64], suggesting these two experience-dependent learning mechanisms might be functionally related.

Short- and long-term learning processes have also been integrated into a single model. Probabilistic sequence-learning models have used separate models with different rules for online learning and retaining observations, before combining them to make rhythmic predictions [41]. Recently, sequence-learning models and PIPPET were combined, such that the expectation template used by PIPPET for real-time phase inference depends on discretized models of (learned) underlying sequence structure [52]. In coupled oscillator models, Hebbian plasticity is proposed to operate along different timescales: short-term transient plasticity enables neural entrainment to a stimulus, while longer-term stable plasticity accounts for perceptual narrowing to culturally familiar rhythms [63]. However, these models addressing multiple timescales of learning have so far seen limited empirical evaluation and comparison.

### 3.4. Interaction with non-temporal and non-auditory features

Rhythm cannot exist without the spectral features of the sounds carrying it (Fig 1D). Neural synchronization [87], perceived tempo [88], and beat [89] all depend on pitch and timbral aspects of sound. The Beyond-the-Beat model [24], an entrainment model that takes the continuous sound signal as input, considers such spectral information: the same rhythm performed on different instruments elicits different output, though melodic and harmonic expectations are not modeled explicitly. In contrast, the probabilistic IDyOM model uses linked viewpoints to represent concurrent expectations for pitch and rhythm [42], but spectral information is discretized into symbolic input. Most models of rhythmic expectations, however, are focused exclusively on timing-related information, taking only event onsets as input. Beyond spectral content, non-auditory modalities—such as visual and vestibular information—may interact with auditory rhythm processing [90,91]. As such, the field’s focus on signals restricted to timing information is a potential major limitation.

### 3.5. Interim summary

In summary, different classes of models capture rhythmic expectations in distinct ways. Entrainment models provide a biologically plausible link between brain mechanisms and rhythmic behavior, flexibly mimicking a wide range of rhythmic phenomena from continuous audio input. However, their large parameter space risks making them descriptive rather than explanatory, and analysis mostly focuses on inferring beat and meter from summary statistics instead of real-time model output. Probabilistic models are well-suited for integrating rhythm with non-temporal features, combining long-term enculturation and short-term adaptation, and potentially unifying metrical and pattern-based expectations. Yet, they lack direct correspondence to underlying neural mechanisms and typically rely on symbolic input or pre-defined templates. Finally, timekeeper models provide a compact framework for describing sensorimotor synchronization, but their narrow focus on motor behavior and isochrony leaves unresolved whether they can explain more complex aspects of rhythm perception.

## 4. Moving rhythm modeling forward

**Fig 2:**
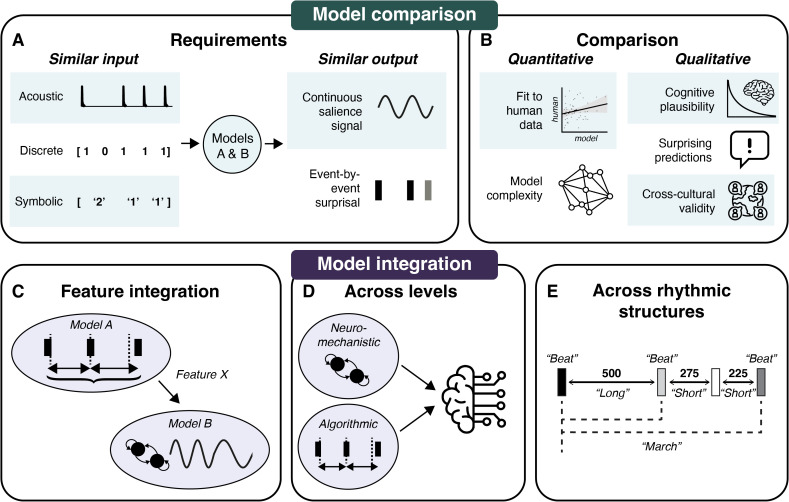
Proposed avenues for moving the field of rhythm modeling forward: model comparison and integration. To compare models (Section 4.1.), input and output signals must be brought into the same space **(A)**. Model comparison may subsequently consider both qualitative and quantitative criteria **(B)**. Ultimately, model integration (Section 4.2.) is a critical step to advance the field, through the integration of features from different classes of models **(C)**, integration of models at different levels of explanation **(D)**, and integration of models that target different rhythmic phenomena **(E)**. Icons were licensed through *Icon Pro* from *The Noun Project* (https://thenounproject.com/).

Given the model classes’ divergent assumptions and foci outlined above, how can we progress? We propose two key avenues: model comparison and integration (Fig 2).

### 4.1. Model comparison

#### 4.1.1. Requirements for comparison.

***4.1.1.1. Similar input.*** Imagine that we want to feed multiple corpora of stimuli through different models to generate sample-wise predictions of rhythmic expectations, to ultimately systematically compare model outputs. We first need to answer two important questions: (1) what type of stimuli do we have (e.g., a symbolic representation, audio), and (2) what type of input is each of our models expecting? While sometimes considered trivial, these questions have important implications for our operationalization of “rhythm” and our interpretation of model results.

While some models operate on raw audio [21,45], many use symbolic representations [41,42,44] or occupy a middle ground where raw audio is converted into a continuous signal representing only onsets [25,48] (Fig 1E). When we compare models operating on symbolic and continuous input, we have to convert continuous data to symbols. For symbols (e.g., A4), the only definable operations are ones of equivalence (e.g., A4! = B4), so we lose spectrotemporal acoustic phenomena, as well as order (e.g., 3 < 5) and scale information (e.g., 4 = 2*2), in the process. The nature of this conversion is therefore crucial. For example, taking inspiration from human categorization [51,78,92], IOIs could be quantized into integer-ratio categories, each with their own symbol. This way, we propose interfacing between real-time and symbolic representations should be cognitively plausible.

***4.1.1.2. Similar output.*** One could propose that we can only directly compare models that target the same aspect of rhythm, at the same level of explanation, with similar output (e.g., “meter in continuous EEG data”). However, regardless of how rhythmic expectations are formed, from a functional perspective, all models of rhythmic expectations should arguably capture something similar: the dynamics of expectations over time [8] that affect behavioral performance and neural excitability [3]. The challenge is therefore to translate the specific construct that is modeled (e.g., neural oscillations, metrical category) to the unifying level of continuous rhythmic expectations.

We see two ways to accomplish this: First, a measure of surprise for each separate event may be extracted from different models, and compared to behavioral or neural data [8] (Fig 2A), as was done for melodic expectations [93]. While this is straightforward for (symbolic) probabilistic models (e.g., using *information content* [42] or *expectancy* [48] at each event or time point), for entrainment models, a non-trivial mapping must be defined from their continuous output to a surprise quantity. Some attempts were made by predicting event times from an oscillator’s phase [40], but the role of amplitude—an important feature of entrainment models [1]—is unclear. One model converted the continuous output signal of an oscillator to a salience measure at discrete time points that indeed correlated with behavior [22], showing promise for bringing continuous output into the same space as surprisal values from symbolic models. A second approach to unifying output is a conversion from discrete or symbolic output to continuous time. For example, metrical categories or symbolic sequences could be mapped onto continuous-time probability distributions [49,51].

#### 4.1.2. Model selection.

***4.1.2.1. Quantitative considerations.*** Once input and output type are harmonized, models can be directly compared, typically by computing goodness-of-fit (GOF) statistics for their fit to empirical data (Fig 2B). While the fit between a computational model and empirical data is considered a necessary starting point, it is not the end point of model evaluation [94]. To prevent overfitting, models of least complexity and flexibility may also be preferred, in terms of both functional form and the number of free parameters [95,96]. Targeted, parsimonious models that describe a small subset of rhythmic behavior could be valuable for understanding specific cognitive processes (e.g., tracking isochronous rhythms) and reduce the risk of overfitting to random noise in the data. However, a model that generalizes to different contexts (e.g., more complex rhythmic patterns) without modifications is preferable. Several criteria have been proposed that quantify the trade-off between GOF and complexity (e.g., the Bayesian information criterion; BIC). More advanced Bayesian model selection approaches treat models as random effects, allowing for the possibility that different individuals may be best described by different models [97–99]. At the group level, these methods quantify the probability that each model is more frequent across participants.

While model comparison based on relative fit and complexity is valuable, it does not guarantee that the ‘winning’ model can actually generate the behavioral effects of interest [100]. To allow for *falsification,* an experiment should be designed that can discriminate between competing models [100]. To this end, experimental stimuli could be selected that show maximally opposing predictions for different models [11].

***4.1.2.2. Qualitative considerations.*** Beyond their fit to empirical data and their complexity, computational models may be judged on their cognitive and biological plausibility [95]. While models should not necessarily implement low-level physiological principles, their computations should be consistent with known features of human cognition. A model of rhythmic expectations should, for example, consider limitations and recency effects in human short-term memory, limits in tapping speed [101], limits in tempo changes [102], and categorization of continuous stimuli [78,92]. Models may also aim for consistency with findings from neuroscience, which have demonstrated the importance of motor regions like the basal ganglia, cerebellum, premotor cortex, and supplementary motor area (SMA) for rhythm processing [70]. Mapping high-level algorithmic processes of computational models onto the neural dynamics and proposed functional role of these regions, like beat predictions based on cyclical firing rate trajectories in the SMA that are selectively sequenced by the striatum [70], is a promising way forward [48,103].

A fourth selection criterion is whether a model makes novel, surprising predictions [9], giving it an edge over models that only predict expected outcomes. In the strongest case, a model predicts a phenomenon that is unexpected from known theories, has not yet been observed, is falsifiable, and confirmed empirically [104]. While novel, surprising predictions strongly support a model, known phenomena may also emerge unexpectedly from its principles. Such emergent behavior, not “built in” by design, also enhances model validity (e.g., [48], where time dilation illusions emerge from first principles of Bayesian inference). This criterium raises the question which rhythmic phenomena are particularly surprising (i.e., unexpected without theoretical insight [9]). One example is the *missing pulse* phenomenon, where rhythms lacking spectral power at the beat frequency still evoke a perceptual and neural response of beat, which is surprising under the assumption of a linear response to rhythmic input [25]. Another potentially “surprising” phenomenon is the human bias toward perceiving rhythms as consisting of categorical durations related by low integer ratios (1:1, 1:2, etc.; [51,78,86,92]). This categorization has been linked to interacting brain oscillations at integer-ratio frequencies [1], but may alternatively result from exposure to rhythms produced by humans whose motor behavior is subject to similar constraints [71].

Finally, models may strive for cross-cultural validity, either by demonstrating generalization across different cultures or populations, thereby showing that the model captures universal aspects of human cognition [86], or by providing explicit mechanisms underlying cultural differences, such as by simulating exposure to different musical corpora [41,51]. Similarly, models should account for individual differences, either by generalizing within a population or by capturing individual differences with meaningful variation in model parameters [31,50].

In short, we suggest that models should be judged as more valuable when they (1) fit empirical data best, (2) make clearly delimited predictions, with the least complexity and flexibility (making a model easier to falsify), (3) are cognitively plausible, (4) make unexpected predictions, and (5) explain cognitive universals, enculturated biases, and individual differences.

### 4.2. Model integration

While comparing models directly is instrumental in scrutinizing and reducing the growing number of rhythm models, a critical next step is to identify how models can be integrated across computational principles and levels of explanation. This could not only lead to a more complete understanding of rhythmic expectations, but would also help assess whether models truly describe distinct cognitive processes or rather describe the same phenomena with different vocabulary (thus aiming to reduce the system to its irreducible parts). Here, we propose three aims for integration: introducing features across models (Fig 2C), integrating models across different levels of explanation (Fig 2D), and integrating across different types of perceived rhythmic structure (Fig 2E).

#### 4.2.1. Adapting features across model types.

Using the strengths of disparate models, we can build composite models that more thoroughly reflect human behavior. One example of integrating features across models comes from the extrapolation of tempo changes forward in time as introduced in timekeeper model ADAM [58]. This feature is crucial for predicting naturalistic rhythm, such as performed music that contains tempo shifts and microtiming. Probabilistic models of melodic expectations [85] and rhythm [11] borrowed this idea by making current event probabilities dependent on recent history. Similarly, an oscillator-based model introduced a dynamic tempo variable that modulates oscillator frequency [10], which led to better fits to behavioral data.

Timekeeper and probabilistic models might also borrow ideas from entrainment models. In one non-linear entrainment model, prediction arises from neural transmission delays inherent in the system (*strong anticipation*; see [72]). These delays reflect the system’s earlier state, allowing a driven system to predict its driver [38]. This is reminiscent of finger taps preceding sounds during synchronization (i.e., *negative mean asynchrony*, though whether this reflects prediction is debated [71,82]). Characteristics of probabilistic models might be improved by incorporating these concepts and making current states dependent on preceding states via a fixed delay. Linking delayed neural coupling to concepts of adaptation and flexibility may also bridge between low-level brain dynamics and cognitive representations, our second aim in integrating models.

#### 4.2.2. Integrating across different levels of explanation.

If cognitive neuroscience aims to link neural processes to cognition and behavior [105], integrating between models at different levels of explanation is particularly important. For example, knowledge can be extrapolated from neurophysiological models to probabilistic predictions at behavioral and cognitive levels. A neural firing rate model that produces self-sustaining oscillations was able to reproduce human psychophysics data showing Bayes-like inference of expected stimulus timing [10]. Similarly, a neural circuit model captured human Bayesian behavior in timing and synchronization tasks [103]. More recently, probabilistic inference of beat phase was shown to be mathematically equivalent to an oscillator model [49]. When a dynamic beat phase estimate and its uncertainty are treated like phase and amplitude (respectively) of a complex variable, the result is an underdamped oscillator with a particular phase-and-amplitude-dependent response to forcing. These examples show the promise of integration between cognitive and neural levels of description.

The behavior-level description of timekeeper models and the neuro-mechanistic description of oscillator models could also potentially be integrated, as both can produce a series of event times showing error-correcting behavior [40,60]. However, certain well-established points of disagreement between the two model classes would have to be overcome, including timekeeper models’ assumptions of linear error correction and one-to-one action-event coupling [40]. The latter is related to the focus of timekeeper models on isochronous sequences, whereas entrainment models also capture beat and meter in non-isochronous rhythm [1,25]. This leads to our final aim in model integration: integrating across different aspects of rhythmic expectations.

#### 4.2.3. Integrating across types of rhythmic expectation.

Whether the cognitive mechanisms for rhythmic expectations depend on task demands [84] and rhythmic structure [80] is an open question. Integrating different types of rhythmic expectations (e.g., beat, pattern) into a unifying model forces us to explicitly define their interactions and task-dependence. Here, bringing the output of different models into the same continuous computational space, agnostic of the modeled structure, may be especially helpful. For example, the continuous probability distribution of PIPPET can incorporate both pattern-based and metrical expectations [52].

If two models with explanatory value cannot be integrated, we have identified irreconcilable ideas that must either be evaluated against each other or cautiously accepted to offer independent cognitive mechanisms. Model integration as such not only allows building better and more overarching models, but also furthers cognitive theory about what rhythmic expectations entail.

## 5. Summary and recommendations

The formal description of rhythmic expectations provided by computational models is crucial to understanding how temporal structure affords benefits in perception, movement, social bonding, and reward associated with musical rhythm [4,5]. Three main classes of computational models (entrainment, probabilistic, and timekeeper models) each have their own focus regarding the level of explanation, type of rhythmic structure, and type of human data they target. Models also deal with learning differently, and non-rhythmic features are rarely accounted for. Often, models of rhythmic expectations are designed to capture specific behavior in response to specific stimuli and work well within this narrow focus.

To reconcile the diverse aims and computational forms of models that arguably target the same underlying concept (i.e., rhythmic expectations), we propose that model comparison and integration are crucial. Model comparison will advance the field by selecting algorithmic descriptions that explain rhythmic behavior best and by forcing us to reduce model flexibility. Model integration can bridge cognitive and neuro-mechanistic explanations, revealing how apparently different rhythmic abilities may result from the same underlying principles. Recent work, such as the PIPPET model, demonstrates how expectations based on rhythmic patterns, beat, and meter can be captured within a unified framework. Alongside PIPPET, models such as the adaptive oscillator model [10] and the neural circuit model [103] show the potential to connect algorithmic accounts (e.g., Bayesian models), low-level neuro-mechanistic processes (e.g., neural oscillations), and high-level theories of auditory-motor coupling in the brain (e.g., the ASAP hypothesis). Such integrative attempts also make it possible to identify truly irreducible abilities, answering pertinent questions about whether multiple mechanisms underlie rhythmic expectations [79,80].

To achieve these goals, we extract several practical recommendations to guide future modeling efforts:

1. *Models to be compared or integrated should handle similar input and produce similar output.* Ideally, models of rhythmic expectations operate on continuous input, or raw audio. Possible conversions of continuous or discrete input to the symbolic domain should consider known cognitive mechanisms involved in rhythm processing, such as categorization. Likewise, model output needs to be brought into the same space, either by extracting a surprise or salience measure for every event in a sequence, or by mapping discrete or symbolic output onto continuous time signals.
2. *Multiple considerations must guide model comparison.* Model evaluation should consider not just GOF, but also model complexity, model scope, cognitive plausibility, unexpectedness of model predictions, and the capacity to capture both cognitive universals and individual differences. Experiments should be designed to maximally differentiate between, and potentially falsify, candidate models.
3. *Model integration must be used to inform theory*. Model integration can not only yield models of greater explanatory power, but also inform theory about irreducible mechanisms of rhythmic expectations.
4. *Open science.* To achieve comparison and integration, models should be publicly available in executable form with clear documentation. Datasets of behavioral and neural data pertaining to rhythmic expectations that may be targeted by models should be made more widely available in open repositories. Frameworks for standardizing and sharing datasets, such as the Cognition and Natural Sensory Processing Initiative (CNSP; https://cnspworkshop.net/) and the Timing Database [106], are essential for promoting reproducibility and integration across research groups.

## 6. Emerging opportunities

Future modeling efforts may broaden the scope of current models by including a wider range of relevant input, like spectral, intensity, and melodic information, and non-auditory information, from visual and tactile modalities. Such efforts may be aided by the availability of rich, multimodal datasets of music and musical behavior [107]. Moreover, while most modeling efforts focus on humans, non-human animals are known to exhibit various aspects of rhythm processing [82]. Cross-species comparisons using the models discussed here may be an especially fruitful way to uncover the mechanisms behind between-species differences in rhythm processing, like differences in beat perception between non-human primates and humans [12,13]. Although entrainment models have been applied to study synchrony in the behavior of non-human animals, such as chorusing in insects, frogs, and birds [108], modeling has rarely been used to directly compare rhythmic behaviors across humans and non-human animals. Notably, one implementation of IDyOM was shown to predict EEG responses to rhythm in rhesus monkeys, similar to humans [67], suggesting that, to some extent, similar processes may underlie rhythmic processing across these species. Extending such modeling approaches to uncover universals and differences across cultures, species, and developmental stages may contribute to the ongoing debate about the evolutionary origins of rhythm processing [5,12], which so far has seen a limited contribution from modeling studies.

Another necessary avenue is to consider deep neural networks [109] and generative AI models [18] for understanding rhythm cognition. Whereas such data-driven models are often designed to optimize metrics of musical quality [110] rather than to understand cognition, there is large merit in using AI to inform theory formation in cognitive psychology [111]. Deep learning models that are trained on large-scale music datasets may implicitly capture statistical regularities, serving as a proxy for human enculturation. Their temporal predictions could be compared with probabilistic models explicitly designed to capture enculturation, such as IDyOM, as well as with human behavioral and neural data [93]. Similarly, the beat synchronization abilities of generative music models [112], models trained on coupled datasets of music and bodily motion such as UniMuMo [113], and deep reinforcement learning models [114], could be compared to human entrainment data. Recent benchmark efforts such as MMAR [115] and MMAU [116] provide large-scale evaluations of audio and music reasoning in multimodal Large Language Models, whereas the MUSE benchmark [117] goes further by directly comparing model performance on tasks like syncopation and meter identification with human perceptual baselines. These comparisons could shed light on whether rhythmic expectations emerge from general-purpose predictive learning or instead require the explicit cognitive mechanisms of probabilistic, entrainment, and timekeeper models. Still, while AI models can serve as theoretical tools, we should be cautious not to assume that human-like performance implies a faithful model of human cognition [111]. Indeed, large AI models often fall short of a key hallmark of cognitive models: interpretability [96].
